# Evidence for Missing Positive Results for Human Papilloma Virus 45 (HPV-45) and HPV-59 with the SPF_10_-DEIA-LiPA_25_ (Version 1) Platform Compared to Type-Specific Real-Time Quantitative PCR Assays and Impact on Vaccine Effectiveness Estimates

**DOI:** 10.1128/JCM.01626-20

**Published:** 2020-10-21

**Authors:** Kahren van Eer, Suzan Leussink, Tim T. Severs, Naomi van Marm-Wattimena, Petra J. Woestenberg, Johannes A. Bogaards, Audrey J. King

**Affiliations:** a National Institute for Public Health and the Environment, Centre for Infectious Diseases, Epidemiology, and Surveillance, Bilthoven, the Netherlands; b Amsterdam UMC, Department Epidemiology & Data Science, Amsterdam, the Netherlands; Cepheid

**Keywords:** human papillomavirus, SPF_10_-DEIA-LiPA_25_, unmasking, TS qPCR assay, vaccination

## Abstract

Human papillomavirus (HPV) epidemiological and vaccine studies require highly sensitive HPV detection systems. The widely used broad-spectrum SPF_10_-DEIA-LiPA_25_ (SPF_10_ method) has reduced sensitivity toward HPV-45 and -59. Therefore, anogenital samples from the PASSYON study were retrospectively analyzed with type-specific (TS) HPV-45 and -59 real-time quantitative PCR (qPCR) assays. The SPF_10_ method missed 51.1% of HPV-45 and 76.1% of HPV-59 infections that were detected by the TS qPCR assays. The viral copy number (VCn) of SPF_10_-missed HPV-45 and -59 was significantly lower than SPF_10_-detected HPV-45 and -59 (*P* < 0.0001 for both HPV types). Sanger sequencing showed no phylogenetic distinction between SPF_10_-missed and SPF_10_-detected HPV-59 variants, but variants bearing the A6562G single-nucleotide polymorphism (SNP) in the SPF_10_ target region were more likely to be missed (*P* = 0.0392). HPV cooccurrence slightly influenced the detection probability of HPV-45 and -59 with the SPF_10_ method. Moreover, HPV-59 detection with the SPF_10_ method was hampered more in nonvaccinated women than vaccinated women, likely due to a stronger masking effect by increased HPV cooccurrence in the former group. Consequently, the SPF_10_ method led to a strong negative vaccine effectiveness (VE) of –84.6% against HPV-59, while the VE based on TS qPCR was 3.1%. For HPV-45, the relative increase in detection in nonvaccinated women compared vaccinated women was more similar, resulting in comparable VE estimates. In conclusion, this study shows that HPV-45 and -59 detection with the SPF_10_ method is dependent on factors including VCn, HPV cooccurrence, and vaccination, thereby showing that knowledge of the limitations of the HPV detection method used is of great importance.

## Abstract (precis)

Human papillomavirus (HPV) epidemiological and vaccine studies require highly sensitive HPV detection systems. The widely used broad-spectrum SPF_10_-DEIA-LiPA_25_ (SPF_10_ method) has reduced sensitivity toward HPV-45 and -59. Therefore, anogenital samples from the PASSYON study were retrospectively analyzed with type-specific (TS) HPV-45 and -59 real-time quantitative PCR (qPCR) assays. The SPF_10_ method missed 51.1% of HPV-45 and 76.1% of HPV-59 infections that were detected by the TS qPCR assays.

## INTRODUCTION

Persistent infection with high-risk human papillomavirus (hrHPV) is associated with multiple types of cancer, including penile, anal, vaginal/vulvar, cervical, and oropharyngeal cancer. Overall, hrHPV was responsible for 690,000 new cancer cases in 2018 (1). Cervical cancer is the most common HPV-related cancer worldwide, and approximately 70% is associated with HPV-16 and -18 (1). An additional 13 types (i.e., HPV-31, -33, -35, -39, -45, -51, -52, -56, -58, -59, -68, -73, and -82) are labeled as hrHPV, and HPV-26, -53, and -66 are considered potentially oncogenic (2).

Prophylactic vaccination offers the best opportunity to reduce the burden of cervical and other HPV-related cancers (3). All currently available HPV vaccines target at least HPV-16 and -18 (4). Nevertheless, accurate monitoring of the other hrHPV types is relevant in postvaccine surveillance, as the remaining hrHPV types determine the residual cancer risk in vaccinated individuals. For example, the cumulative attribution of HPV-45 and -59 to cervical cancer globally compared to other (potential) hrHPV is 5.9% and 1.1%, respectively (5).

The broad-spectrum L1-based SPF_10_-LiPA_25_ version 1 method (DDL Diagnostics, the Netherlands), here called the SPF_10_ method, is widely used in epidemiological HPV monitoring and vaccine effectiveness studies. This test utilizes a cocktail of primers to detect many of the essential hrHPV types, such as HPV-16, -18, -31, -33, -35, -39, -45, -51, -52, -56, -58, and -59. Furthermore, the use of generalized primers to target a broad range of HPV types reduces the total number of primers needed (6, 7).

One limitation of the SPF_10_ method is the various HPV detection sensitivities. The limit of detection (LOD) for the included hrHPV types, as determined by the manufacturer, is lowest for HPV-18 and -33 with one DNA copy per reaction. Conversely, the LOD is highest for HPV-59 with 44 DNA copies per reaction, followed by HPV-45 with 9 DNA copies per reaction.

Recent observational studies using the SPF_10_ method in Dutch sexual health center visitors reported high vaccine effectiveness (VE) against HPV-16 and -18 and significant cross-protection against HPV-31, -35, -45, and -52. Conversely, remarkably strong negative VE against HPV-59 was also estimated (8, 9). In light of these observations, the highly negative VE against HPV-59 was the motivation behind the in-depth analysis of HPV-59-specific detection sensitivity of the SPF_10_ method. Furthermore, the HPV-45-specific LOD of the SPF_10_ method is relatively high. Therefore, SPF_10_ detection rates of these two types and the reliability of associated VE estimates require further investigation.

Here, we compared the detection rate of HPV-45 and -59 with the SPF_10_ method with two separate HPV-45 and -59 type-specific (TS) quantitative PCR (qPCR) assays in anogenital swabs of Dutch sexual health center visitors, previously described in Woestenberg et al. (8). We also examined the effect of several virological factors influencing HPV-45 and -59 detection with the SPF_10_ method. Additionally, the HPV-45- and HPV-59-specific VE estimates were calculated based on the detection rates of the SPF_10_ method and the TS qPCR assays.

## MATERIALS AND METHODS

### Clinical samples.

For the current research, genital and anal swab samples from men and women who participated in 2009, 2015, and 2017, were selected from the biennial cross-sectional PASSYON (PApillomavirus Surveillance among STI clinic YOungsters in the Netherlands) study. The study design is described in detail elsewhere (10). Briefly, the PASSYON study started in 2009, before the introduction of the Dutch HPV vaccination program. Male and female sexual health center visitors, 16 to 24 years of age, were asked to fill in a questionnaire including vaccination status and to provide a self-collected genital swab for HPV testing. A subset of the participants also provided an anal swab. This study was approved by the Medical Ethical Committee of the University of Utrecht, the Netherlands (protocol number 08/397).

### Sample preparation and SPF_10_-based HPV genotyping.

Anal and genital swabs were stored at –20°C until analysis. Then, a 200-μl specimen was spiked with Phocine herpesvirus-1 (PhHV-1) prior to DNA extraction using the MagNA Pure 96 (MP96) platform (total nucleic acid isolation kit, Roche Diagnostics, Rotkreuz, Switzerland). The DNA was eluted in 100 μl elution buffer. All DNA samples were tested for HPV types using the HPV SPF_10_ LiPA_25_ version 1 method according to the manufacturer’s protocol (DDL Diagnostic Laboratory, the Netherlands). Briefly, HPV DNA was amplified using the broad-range SPF_10_ primers. Subsequently, a DNA enzyme immunoassay (DEIA) was used for the detection of HPV DNA in all samples. In order to determine HPV genotypes, amplicons of all HPV DNA-positive samples were analyzed in the line probe assay (LiPA), which is able to identify 25 HPV genotypes, including hrHPV-16, -18, -31, -33, -35, -39, -45, -51, -52, -56, -58, -59, and -68 and potential hrHPV-66. The reproducibility of the SPF_10_ method was analyzed and met the criteria of >90% identical or compatible HPV types. Validation of the SPF_10_ method with DNA isolated with the MP96 compared to the MagNA Pure LC 2.0 was successfully performed with >92% identical genotyping results.

### Type-specific real-time qPCR (TS qPCR).

The viral copy number (VCn) of all DNA samples, previously isolated with the MP96, was quantified with separate HPV-45 and HPV-59 type-specific (TS) real-time quantitative PCR (qPCR) assays. TS qPCR assays for HPV-45 and -59 were developed in previous research (11, 12). Each TS qPCR assay targets a region on the capsid-encoding L1 gene and is adjusted for specificity from Seaman et al. (13) and is optimized to exceed sensitivity levels of the SPF_10_ method. For HPV-45 and -59, primers and probes were designed to target a 107-bp and a 116-bp region within the L1 gene of HPV-45 and -59, respectively (12). Briefly, 5 μl DNA was added to 15 μl master mix containing LightCycler 480 Probes Master (Roche), 400 nM forward primer, 400 nM reverse primer, and 100 nM probe. The TS qPCR assay was performed on the Roche LightCycler 480 platform (Roche) with cycling conditions of 95°C for 10 min, followed by 50 cycles of 95°C for 15 s and 60°C for 30 s. The specificity and sensitivity of the TS qPCRs for HPV-45 and -59 were determined previously as described by van der Weele et al. (12). Table S1 in the supplemental material shows the differences between the SPF_10_ method and the HPV-45 and -59 TS qPCR assays.

### Phylogenetic and SNP analyses of HPV-59 L1 sequences.

Sanger sequencing was performed on the L1 gene of a subset of HPV-59 SPF_10_ and/or TS qPCR positive samples. Prior to sequencing, the complete L1 gene was PCR amplified in three overlapping fragments using the primers listed in Table S2. PCR amplification and Sanger sequencing were performed similarly as described for the HPV16/18 L1 gene (14). Sequence assembly was carried out against the reference HPV-59 isolate (GenBank accession number X77858) retrieved from the Papilloma Virus Episteme (PAVE) database (15; https://pave.niaid.nih.gov/). Phylogenetic and single nucleotide polymorphism (SNP) analyses were performed with BioNumerics 7.6.3. Additionally, nine HPV-59-positive samples detected by the TS qPCR only, with a VCn above the SPF_10_ detection limit for HPV-59, were confirmed by an external lab with the SPF_10_ method (20 μl SPF_10_ product taken for LiPA instead of the standard 10 μl), an alternative highly sensitive MPTS2 PCR Luminex assay (16), and next-generation sequencing (NGS). Three, seven, and six samples were found to be HPV-59 positive after SPF_10_ genotyping, the PCR Luminex assay, and NGS, respectively.

### Study population to measure VE estimates.

In order to evaluate the potential change in HPV-45- and HPV-59-specific VE estimates, HPV-45 and -59 detection rates were analyzed with the SPF_10_ method and the TS qPCR assays in vaccinated and nonvaccinated women. For this analysis, we selected genital samples from women eligible for vaccination (born in 1993 or later) and with a known self-reported vaccination status.

### Statistical analyses.

Statistical analysis was performed with R Studio 3.6.0 and GraphPad Prism 8. Cohen’s κ statistic was used to assess the degree of agreement between the SPF_10_ method and the TS qPCR assays. κ agreement levels are stated according to reference 17. In addition, McNemar’s test was used for pairwise comparison of the HPV-45 and -59 positivity rates with the SPF_10_ method or the TS qPCR assays. The Mann-Whitney U (MWU) test was used to compare VCn measurements between SPF_10_-detected and SPF_10_-missed HPV-45 and -59 given in copies (c) per reaction (rxn). Only VCn-positive measurements were included. Fisher’s exact test was used to analyze potential differences in the presence of SNPs in SPF_10_-missed and SPF_10_-detected HPV-59 variants. Only the SNPs occurring in more than one sample were taken along in the analysis. The effect of cooccurring HPV types on the HPV-45 and -59 SPF_10_ detection rate within TS qPCR positive samples was analyzed with a logistic regression model and corrected for VCn. VE was estimated as 1 – adjusted odds ratio (OR) × 100% according to Woestenberg et al. (8) and corrected for known demographics and sexual behavior with a logistic mixed model. A *P* value smaller than 0.05 was considered statistically significant.

### Data availability.

The sequences obtained during this study have been submitted to GenBank (accession numbers MT934137 to MT934364).

## RESULTS

### HPV-45 and -59 detection with the SPF_10_ method and TS qPCR assays.

**FIG 1:**
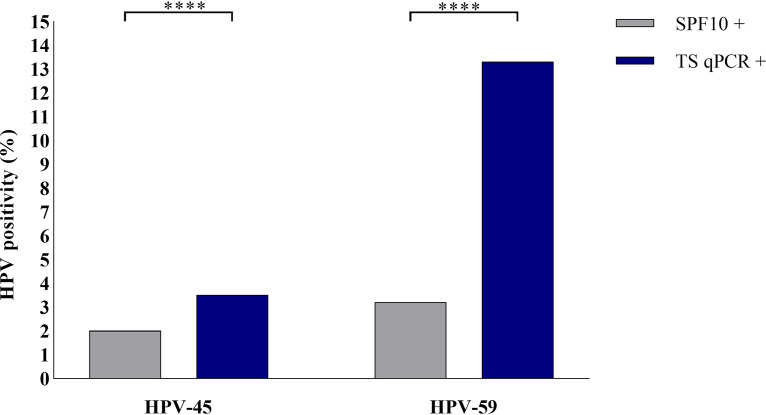
HPV-45 and -59 positivity rate as determined by the SPF_10_ method and the TS qPCR assays. ****, *P* < 0.0001.

For this study, 5,944 genital and anal swab specimens were previously tested with the SPF_10_ method. In total, 2.0% (*n* = 113) and 3.2% (*n* = 190) of the specimens were HPV-45 and HPV-59 positive, respectively. Reanalysis of all samples with the TS qPCR assays identified 1.7% (*n* = 109) and 10.1% (*n* = 598) additional swab specimens as HPV-45 and -59 positive, respectively. Sporadically, the SPF_10_ method detected HPV-45 (0.2%, *n* = 9) and HPV-59 (0.03%, *n* = 2) positivity not detected by the TS qPCR assays. The agreement between the SPF_10_ method and the TS qPCR assay was “substantial” (κ, 0.63; 95% confidence interval [CI], 0.57 to 0.69) for HPV-45 but only “fair” (κ, 0.35; 95% CI, 0.31 to 0.39) for HPV-59 (Table 1). Additionally, the results indicate a significant difference in HPV-45 (*P* < 0.0001) and HPV-59 (*P* < 0.0001) detection rate between the TS qPCR assays and the SPF_10_ method on sample level (Fig. 1).

**TABLE 1:** Overview of the HPV-45 and -59 positivity rate as determined by the SPF_10_ method and TS qPCR assays^*a*^

HPV	SPF_10_ result	No. of TS qPCR + (%)	No. of TS qPCR – (%)	Total (%)	Kappa (95% CI)^*b*^	McNemar’s *P*
45	SPF_10_ +	104 (1.8)	9 (0.2)	113 (2.0)	0.629 (0.567–0.691)	<0.0001
	SPF_10_ –	109 (1.7)	5,722 (96.3)	5,831 (98.0)		
	Total	213 (3.5)	5731 (96.5)	5,944 (100)		
59	SPF_10_ +	188 (3.2)	2 (0.0)	190 (3.2)	0.352 (0.314–0.389)	<0.0001
	SPF_10_ –	598 (10.1)	5,156 (86.7)	5,754 (96.8)		
	Total	786 (13.3)	5,158 (86.7)	5,944 (100)		

a +, positive; –, negative.

b Kappa values are stated with a 95% confidence interval.

### HPV-45 and -59 VCn and SPF_10_ detection rate.

**FIG 2:**
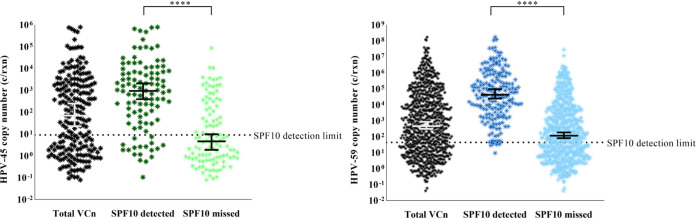
HPV-45 (left) and -59 (right) log VCn measurements of detected and missed infections with the SPF_10_ method. ****, *P* < 0.0001.

Since the LOD of the SPF_10_ method influences the HPV detection sensitivity, HPV-45 and -59 VCn was compared between samples detected or missed by the SPF_10_ method (Fig. 2, left and right, respectively). The median VCn of detected HPV-45 (947.5 c/rxn, *n* = 104) was significantly higher (*P* < 0.0001) than the median VCn of missed HPV-45 (4.64 c/rxn, *n* = 109). Similarly, the median VCn of detected HPV-59 (42,700 c/rxn, *n* = 188) was significantly higher (*P* < 0.0001) than the median VCn of missed HPV-59 (116 c/rxn, *n* = 598). Thus, SPF_10_-detected HPV-45 and -59 generally have a higher VCn than SPF_10_-missed HPV-45 and -59, although (non)detection did not always coincide with the LOD as determined by the manufacturer.

### Sequencing of HPV-59 and L1 variant analysis.

The difference between the detection rates of the TS qPCR assays and the SPF_10_ method was stronger for HPV-59 than HPV-45 and served as the incentive to further analyze the authenticity of the HPV-59-positive samples. L1 sequencing was performed on a subset of HPV-59-positive samples (*n* = 228), which confirmed the presence of HPV-59. Of this subset, 43% (*n* = 99) were determined HPV-59 positive with the SPF_10_ method and TS qPCR, and 57% (*n* = 129) were determined HPV-59 positive with only the TS qPCR. All sequenced samples had an HPV-59 VCn above the LOD of 44 c/rxn, ranging from 51.4 to 175 × 10^6^ c/rxn, with the exception of one sample with a VCn of 33.7 c/rxn. Furthermore, the sequences were dichotomized into variant lineages A or B, based on Chen et al. (18). Lineage B (82%, *n* = 187) was more abundant than lineage A (18%, *n* = 41), although no phylogenetic distinction between the SPF_10_-detected and SPF_10_-missed HPV-59 variants was found (Fig. S1). However, HPV-59 variants harboring any SNPs within the SPF_10_ primer target region of L1 were significantly more likely to be missed (*P* = 0.0027) by the SPF_10_ method compared to wild-type HPV-59 variants (100% similarity to the SPF_10_ target region) (Table 2). Specifically, HPV-59 variants bearing the A6562G SNP were significantly more likely to be missed by the SPF_10_ method (*P* = 0.0392). Overall, the results indicate that detected and missed HPV-59 variants are phylogenetically similar, but missed HPV-59 variants more often carry the A6562G SNP than do detected HPV-59 variants.

**TABLE 2:** Overview of the SNPs in the SPF_10_ target region of the HPV 59 L1 gene (5,606–7,132 bp) from reference strain X77858

SNP	SPF_10_ missed (%)	SPF_10_ detected (%)	Fisher’s exact *P* value
Total	129 (100)	99 (100)	
Any SNP	28 (21.7)	7 (7.1)	0.0027
Wild type	101 (78.3)	92 (92.9)	
T6553C	14 (10.9)	5 (5.1)	0.1487
Wild type	115 (89.1)	94 (94.9)	
A6562G	14 (10.9)	3 (3.0)	0.0392
Wild-type	115 (89.1)	96 (97.0)	

### The effect of HPV cooccurrence on HPV-45 and -59 detection.

Cooccurrence of HPV types may hamper HPV-45 and -59 detection with the SPF_10_ method. After correcting for the effect of VCn, HPV-45 detection probability increased with additional HPV types, although not consistently with increasing number of cooccurring HPV types (Table 3). Nevertheless, the odds of HPV-45 detection significantly increased in a coinfection with one additional HPV type relative to single infections (OR, 3.87; 95% CI, 1.24 to 12.1). After pooling the samples with cooccurring HPV types, the odds of HPV-45 detection relative to single HPV-45 were 1.61 (95% CI, 0.67 to 3.88). Conversely, the probability of detecting HPV-59 consistently decreased with additional HPV types (Table 3), although this decrease was only significant with three additional HPV types (OR, 0.32; 95% CI, 0.15 to 0.70). After pooling the samples with cooccurring HPV types, the odds of HPV-59 detection relative to single HPV-59 were 0.60 (95% CI, 0.32 to 1.11). Overall, cooccurring HPV types slightly increased HPV-45 detection probability and slightly decreased HPV-59 detection probability, although this effect was not always statistically significant.

**TABLE 3:** Numbers of SPF_10_ detected (+) and SPF_10_ missed (–) HPV-45 and -59 in single infections (0), with 1 to ≥5 cooccurring HPV types and pooled samples with cooccurring HPV types (pooled)^*a*^

HPV type	HPV-45			HPV-59		
	SPF_10_ – (%)	SPF_10_ + (%)	OR (95% CI)	SPF_10_ – (%)	SPF_10_ + (%)	OR (95% CI)
0	21 (19.3)	12 (11.5)	Reference	79 (13.2)	22 (11.7)	Reference
1	14 (12.8)	24 (23.1)	3.87 (1.24–12.1)	129 (21.6)	44 (23.4)	0.70 (0.34**–**1.42)
2	24 (22)	20 (19.2)	0.95 (0.32**–**2.83)	127 (21.2)	49 (26.1)	0.89 (0.44**–**1.79)
3	23 (21.1)	17 (16.4)	1.32 (0.44**–**3.97)	127 (21.2)	24 (12.8)	0.32 (0.15–0.70)
4	15 (13.8)	14 (13.5)	1.23 (0.37**–**4.06)	70 (11.7)	24 (12.8)	0.56 (0.25**–**1.26)
≥5	12 (11)	17 (16.3)	1.97 (0.59**–**6.57)	66 (11)	25 (13.3)	0.45 (0.20**–**1.03)
Pooled	88 (80.7)	92 (88.5)	1.61 (0.67**–**3.88)	519 (86.8)	166 (88.3)	0.60 (0.32**–**1.11).

a Odds ratios (ORs) state the odds of HPV-45 or -59 detection in samples with cooccurring HPV types relative to single infections, corrected for VCn.

### Impact of the detection method on vaccine effectiveness estimates.

**FIG 3:**
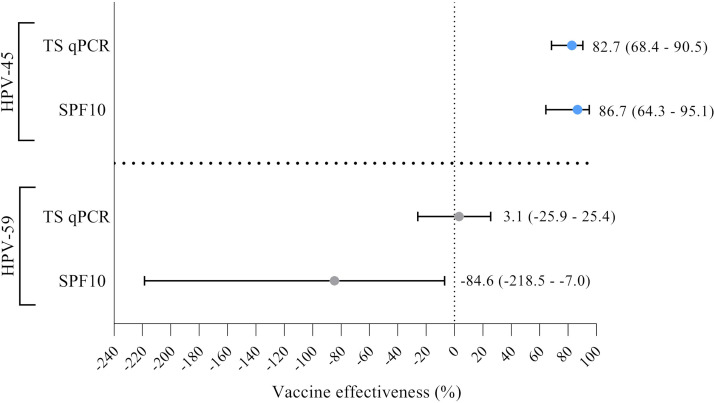
HPV-45 and -59 vaccine effectiveness (VE) estimates and 95% confidence intervals with the SPF_10_ method and the TS qPCR assays after adjusting for confounders (sexual risk, behavior, and demographics).

HPV-45 and -59 detection rates among women eligible for vaccination (*n* = 1,820) were analyzed with the SPF_10_ method and the TS qPCR assays (Table 4). Of the eligible women, 63.4% (*n* = 1,153) were vaccinated and 36.6% (*n* = 667) were nonvaccinated. The SPF_10_ method and the TS qPCR detected significantly more HPV-45 in nonvaccinated women (3.1% [*n* = 21] and 7.0% [*n* = 47], respectively) than vaccinated women (0.5% [*n* = 6] and 1.3% [*n* = 16], respectively) (*P* < 0.0001 for both tests). The adjusted SPF_10_-based VE estimate against HPV-45 of 86.7% (95% CI, 64.3 to 95.1) was comparable to the TS qPCR-based VE estimate of 82.7% (95% CI, 68.4 to 90.5). Interestingly, the SPF_10_ method detected significantly more HPV-59 (*P* = 0.017) in vaccinated women (5.2%, *n* = 61) than in nonvaccinated women (2.8%, *n* = 19). The TS qPCR detected similar HPV-59 positivity (*P* = 0.902) in nonvaccinated women (19.0%, *n* = 127) and vaccinated women (19.3%, *n* = 223). The adjusted SPF_10_-based VE estimate against HPV-59 was strongly negative at –84.6% (95% CI, –218.5 to –7.0), but this changed to 3.1% (95% CI, –25.9 to 25.4) with the TS qPCR (Fig. 3). To further investigate the effect of HPV cooccurrence on the VE against HPV-59 as estimated by the SPF_10_ method, the analyses of cooccurrence on HPV-59 detection by the SPF_10_ method in relation to vaccination status were repeated. In general, HPV-59 detection probability was increased in vaccinated compared to nonvaccinated women in the presence of HPV cooccurrence (OR, 2.38). Conversely, no big differences between the detection probability of single HPV-59 in vaccinated compared to nonvaccinated women (OR, 0.89) was observed.

**TABLE 4:** HPV-45 and -59 positivity rate with the SPF_10_ method and the TS qPCR assays among vaccinated and nonvaccinated women

HPV type^*a*^	SPF_10_			TS qPCR		
	Vaccinated (%)	Not vaccinated (%)	*P* value^*b*^	Vaccinated (%)	Not vaccinated (%)	*P* value^*b*^
45 +	6 (0.5)	21 (3.1)	**<0.0001**	16 (1.3)	47 (7.0)	**<0.0001**
45 –	1,147 (99.5)	646 (96.9)		1,137 (98.7)	620 (93.0)	
Total	1,153 (100)	667 (100)		1,153 (100)	667 (100)	
59 +	61 (5.2)	19 (2.8)	**0.017**	223 (19.3)	127 (19.0)	0.902
59 –	1,092 (94.8)	648 (97.2)		930 (80.7)	540 (81.0)	
Total	1,153 (100)	667 (100)		1,153 (100)	667 (100)	

a +, positive; –, negative.

b A *P* value in bold indicates a significant difference.

## DISCUSSION

Insight into type-specific HPV prevalence and the effectiveness of HPV vaccination relies on robust, accurate, and reliable HPV detection. The SPF_10_ method is an established standard and is commonly used in epidemiological and vaccine effectiveness studies for type-specific HPV monitoring (19–23). The assay targets a part of the HPV L1 sequences, permitting simultaneous amplification of over 50 HPV genotypes in a single test (6, 7). Here, we compared the HPV-45 and -59 detection rate of the widely used SPF_10_ method with two type-specific qPCR assays. We showed that in anogenital samples from young Dutch visitors of sexual health centers, the SPF_10_ method is hampered in particular for HPV-59 detection and to a lesser extent for HPV-45 detection compared to the TS qPCR assays. Consequently, the SPF_10_ method underestimates HPV-45 and -59 prevalence, affecting HPV-45 and -59 VE estimates to various degrees. Additionally, we provided insight into the virological factors that may influence SPF_10_ detection.

The detection sensitivity of the SPF_10_ method was especially diminished for HPV-59 but also for HPV-45, missing 76.1% (598/786) and 51.1% (109/213) of TS qPCR assay positive results, respectively. In contrast, the TS qPCR assays missed only 8% (9/113) of SPF_10_ HPV-45-positive results and 1.1% (2/190) of SPF_10_ HPV-59-positive results. The lower HPV-45 and -59 detection limit of the TS qPCR compared to the SPF_10_ method is emphasized by the significantly higher VCn of SPF_10_-detected HPV-45 and -59 compared to the VCn of SPF_10_-missed HPV-45 and -59. Similarly, previous research reported that HPV-16, -18, and -52 with low viral load was often missed by the clinically validated GP5+/6+ HPV detection assay, while being detected by the more sensitive SPF_10_ method (24). Furthermore, Clifford et al. reported on the impact of low VCn on the HPV detection rate of the broad-range GP5+/6+-based PCR compared to the multiplex type-specific E7-MPG. Nevertheless, missing HPV-53, -59, and -82 was likely due to less-than-optimal PCR primer alignment (25). Contrary to Clifford et al., we observed a VCn-dependent HPV-59 detection rate with the SPF_10_ method. Thus, differences between the sensitivity of various HPV detection tests can be caused by various issues, including virology (i.e., VCn for the SPF_10_ method) and technical limitations (i.e., suboptimal primer alignment for the GP5+/6+-based PCR).

Interestingly, the VCn of many SPF_10_-missed HPV-59 and several HPV-45-positive samples was above the SPF_10_ LOD. Nonetheless, the generated HPV-59 L1 sequences (VCn above the LOD) suggest that TS qPCR-detected HPV-59 positives are genuine. Phylogenetic differences and/or the presence of SNPs might alter primer alignment efficiency, explaining the detection failure of HPV-59 with a VCn above the SPF_10_ LOD. Although no phylogenetic distinction was found between L1 sequences of SPF_10_-detected and -missed HPV-59 variants, more missed HPV-59 variants carried the A6562G SNP. A6562G is located 11 bases from the beginning of the SPF_10_ target region, possibly causing less efficient SPF_10_ primer binding. Detection failure due to primer misalignment has been observed previously for HPV-53, -59, and -82 detection with the GP5+/6+ assay (25). Likewise, GP5+/6+-impaired primer hybridization on HPV-52 was found due to a synonymous T6764C substitution (26). However, many HPV-59-positive results with a VCn above the LOD and with no variation in the SPF_10_ target region were still missed, suggesting that additional factors influence SPF_10_ detection performance.

Detection of HPV-45 and -59 may be hampered by cooccurring HPV types competing for PCR reagents, a process called masking. The SPF_10_ method’s use of generalized primers, which permits simultaneous amplification of multiple HPV types, increases the risk of detection failure by masking compared to the TS qPCR assays, which use type-specific primers (27–29). Our results show a trend in which SPF_10_ detection of HPV-45 and -59 is positively and negatively impacted by HPV cooccurrence, respectively. The varying effect of HPV cooccurrence on HPV-45 and -59 detection could be partially explained by the difference in the detection sensitivity of the SPF_10_ method. We corrected for VCn of HPV-45 and -59 but lacked information on other confounders (e.g., VCn of cooccurring HPV types). Moreover, individual factors (e.g., sexual risk behavior) or sample characteristics might explain why detection of one HPV type increases the chance of detecting HPV-45 with the SPF_10_ method. As a result, the impact of HPV cooccurrence is different for HPV-45 and -59 detection by the SPF_10_ method.

HPV vaccination reduces the prevalence of vaccine-targeted HPV-16 and -18 but also several nonvaccine HPV types, including HPV-45 (8). Recent studies reported that HPV-45 prevalence among 16- to 24-year-old women in the UK ranged from 2.6 to 4.2% in 2008 (prevaccination) and decreased to 0.2 to 0.9% in 2016 (postvaccination) (30, 31). A Finnish cohort study reported similar HPV-59 prevalence in vaccinated women and nonvaccinated women, ranging between 1.1 and 9.9% and 0 and 9.5%, respectively (32). The studies conducted in the UK and Finland used alternative HPV detection methods. We show that the SPF_10_ method-based HPV-45 and -59 prevalence in vaccinated and nonvaccinated women was within the range of that observed in the Finnish and UK studies. Nevertheless, the SPF_10_ method underestimated HPV-45 and -59 prevalence in both vaccinated and nonvaccinated women compared to the TS qPCR assays. For HPV-45, the SPF_10_ method and the TS qPCR showed a similar relative increase in prevalence in vaccinated women and nonvaccinated women, resulting in comparable VE estimates. Interestingly, the TS qPCR assay showed a stronger relative increase in HPV-59 prevalence in nonvaccinated women compared to vaccinated women, alleviating the highly negative VE estimate obtained by the SPF_10_ method. In fact, the TS qPCR assay found similar HPV-59 prevalence among vaccinated and nonvaccinated women, leading to a negligible VE estimate. Previous studies also reported relative increases of HPV-45, -58, and -59 persistent infections among the nonvaccinated group compared to the vaccinated group with the MPTS12 RHA detection assay instead of the SPF_10_ method, resulting in a large increase in VE (33). Therefore, the use of alternative HPV detection methods in addition to or instead of the SPF_10_ method to monitor postvaccine HPV prevalence and estimate VE may be preferred for HPV types with relatively high LOD values, such as HPV-45 and -59, but may also be worth investigating for other HPV types.

The discrepant HPV-59 positivity rates determined by the SPF_10_ method and the TS qPCR assay suggest unmasking rather than type replacement. Type replacement is the genuine increase of nonvaccine HPV types due to a reduction in vaccine-targeted HPV types after vaccination. So far, no evidence for HPV type replacement has been found, possibly due to limited follow-up since the widescale implementation of HPV vaccination (32, 34, 35). Unmasking causes removal of vaccine-targeted HPV types competing for PCR reagents, leading to detection of HPV types previously less visible, especially types with low concentrations (21, 28, 33). Our findings indicate that the higher HPV-59 prevalence in vaccinated women than nonvaccinated women determined by the SPF_10_ method is due to unmasking. Moreover, we found that HPV-59 was missed more often in nonvaccinated women than vaccinated women with the SPF_10_ method in the presence of cooccurring HPV types. These findings suggest that HPV-59 detection is facilitated in vaccinated women due to the (cross-)protective effects of the vaccine. Vaccinated women carry fewer coexisting HPV types with possibly lower viral load compared to nonvaccinated women (12), thereby unmasking previously less visible HPV types.

Our study has several strengths and limitations. Our study population is at high risk of HPV infection, possibly generating a higher HPV-45 and -59 prevalence compared to general populations. Vaccination status was self-reported, but previous research indicated that recall bias is likely to be limited (8). HPV genotyping using the SPF_10_ LiPA requires the hybridization of specific probe lines and can require more than one probe line to confirm the presence of certain HPV types (for example, HPV-31). Furthermore, HPV-68 and -73 cannot be discriminated from each other. Nevertheless, these complications do not apply for HPV-45 and -59, which require single probe lines. VCn measurements are not normalized for cellular content, e.g., by quantifying β-actin with a qPCR. However, the main goal of this study was to examine the detection failures of the SPF_10_ method in a reaction. Furthermore, retrospective testing of HPV-45 and -59 with the TS qPCR assays might have been affected by the decrease in HPV-45 and -59 DNA concentrations over time. Nevertheless, this analysis emphasizes the high detection sensitivity of TS qPCR assays, as significantly more HPV-45 and -59 was detected in relatively old samples. Also, we did not perform VCn quantifications for the cooccurring HPV types. Although beyond the scope of this article, VCn quantifications will be necessary to perform in-depth analyses of the effect of HPV cooccurrence on HPV-45 and -59 detection with the SPF_10_ method.

In summary, we demonstrate that SPF_10_ detection performance is limited mostly for HPV-59 and to a lesser extent for HPV-45 compared with the TS qPCR assays. Suboptimal detection by the SPF_10_ method is influenced by low VCn, intratype genetic variation, and HPV cooccurrence. Use of the SPF_10_ method to monitor HPV-45 and -59 may lead to inaccuracies, especially in study populations varying in HPV prevalence, vaccination coverage, and amount of cooccurring HPV types. For HPV-59, specifically, differences in detection performance of the SPF_10_ method compared to the TS qPCR assay resulted in the change of a highly negative VE estimate to a negligible vaccination effect. Consequently, HPV-45 and -59 detection with the SPF_10_ method alone is insufficient and should be combined with a TS qPCR assay in order to improve the accuracy. Understanding the limitations of the HPV detection method used within a specific study population is crucial for correct test usage in epidemiological studies and vaccine surveillance.

## Supplementary Material

Supplemental file 1
