# Supplementary material for: Evidence for Missing Positive Results for Human Papilloma Virus 45 (HPV-45) and HPV-59 with the SPF10-DEIA-LiPA25 (Version 1) Platform Compared to Type-Specific Real-Time Quantitative PCR Assays and Impact on Vaccine Effectiveness Estimates
Source: J Clin Microbiol. 2020 Oct 21;58(11):e01626-20. doi: 10.1128/JCM.01626-20 (PMC7587105; doi:10.1128/JCM.01626-20)

SUPPLEMENTARY INFORMATION

Table S1. Overview of the differences between the SPF_{10}-DEIA-$LiPA_{25}$ (SPF_{10} method) and the type-specific qPCR.

**Characteristics**	**SPF_{10} method**	**Type-specific qPCR**
**HPV detection**	Broad-spectrum	Type specific
**HPV amplicon size L1**	65 bp	107 bp/116 bp [c]
**PCR system**	Block PCR	Real-time qPCR
**PCR volume**	50µl (10µl sample DNA)	15µl (5µl sample DNA)
**Detection limit (copies per reaction)**	9 / 44 [a]	1.8/1.2[b]

[a] Detection limit HPV-45 and -59, respectively (LOD as determined by manufacturer).

[b] Detection limit for HPV-45 and -59, respectively. Retrieved from P. van der Weele, E. van Logchem, P. Wolffs, I van den Broek, M. Feltkamp, H de Melker, CJLM Meijer, H. Boot and A.J. King (J. Clin. Virol 83:6-11, 2016).

[c] PCR product size for HPV-45 and -59, respectively.

Table S2. HPV 45 and 59 forward (Fw) and reverse (Rv) primer sequences, which amplify L1 in three fragments (A,B, and C) and their respective working concentrations.

Oligonucleotide	Fw sequence (5' → 3')	Rv sequence (5' → 3')	Solution	Product
**HPV45-L1-A**	ACAGTACCATTAACATCTGCAT	TTACAAAGTGTGCCCTTGG	10µM	719bp
**HPV45-L1-B**	GGGCAGCCTTTAGGTATTG	TAGTACTGCGGGTAGTGTC	10µM	681bp
**HPV45-L1-C**	TGTGTATTCCCCTTCTCCC	ACAGTAACACCATACATGCC	10µM	790bp
**HPV59-L1-A**	GCCTGGGATGTTCCTGTAAA	ACAGCAGATGCTACATGAGA	10µM	580bp
**HPV59-L1-B**	GTAGGTGTTGAAATCGGTCG	AGAAAGATTGGTGCTGCG	10µM	705bp
**HPV59-L1-C**	GTGGGTCTGTGGTTACTTCT	TGCAACCACACATGAAACTG	10µM	779bp
**HPV59-L1-full***	GCCTGGGATGTTCCTGTAAA	TGCAACCACACATGAAACTG	10µM	1852bp

*If VCn is >10,000 c/rxn the HPV59-L1-fullPCR is used.

FIG S1. Maximum parsimony analysis of the HPV-59 L1 sequences (n=228), either detected by the SPF_{10} method and the TS qPCR (n=99) or detected by the TS qPCR and missed by the SPF_{10} method (n=129).

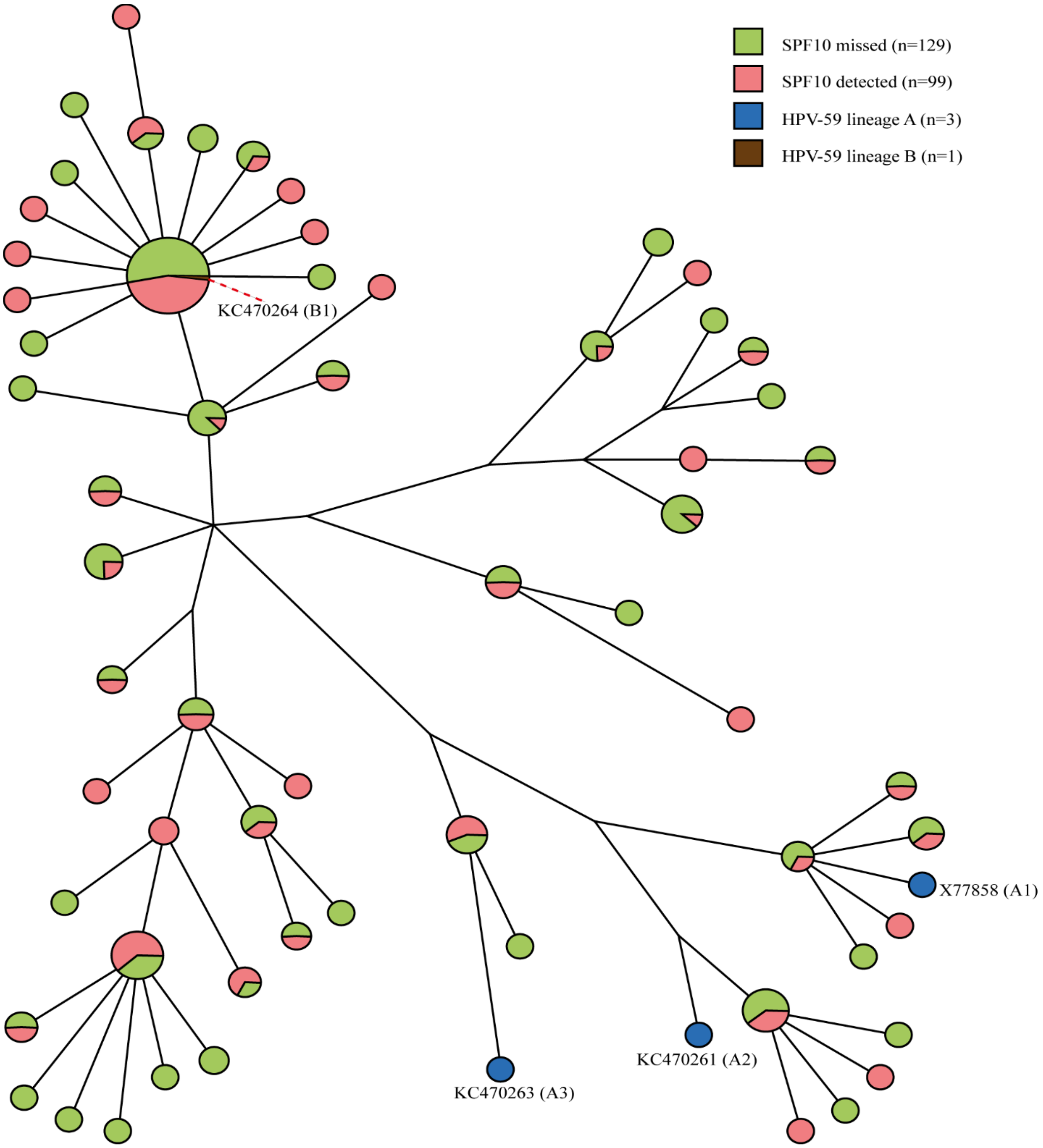

Supplement: Supplemental file 1 [file JCM.01626-20-s0001.pdf]
